# Preliminary Study on the Optimization of Femtosecond Laser Treatment on the Surface Morphology of Lithium Disilicate Glass-Ceramics and Highly Translucent Zirconia Ceramics

**DOI:** 10.3390/ma15103614

**Published:** 2022-05-18

**Authors:** Masanao Inokoshi, Kumiko Yoshihara, Masayuki Kakehata, Hidehiko Yashiro, Noriyuki Nagaoka, Watcharapong Tonprasong, Kaiqi Xu, Shunsuke Minakuchi

**Affiliations:** 1 Department of Gerodontology and Oral Rehabilitation, Graduate School of Medical and Dental Sciences, Tokyo Medical and Dental University, Tokyo 113-8549, Japan; tonprasong.gerd@tmd.ac.jp (W.T.); xugerd@tmd.ac.jp (K.X.); s.minakuchi.gerd@tmd.ac.jp (S.M.); 2 National Institute of Advanced Industrial Science and Technology (AIST), Health and Medical Research Institute, Takamatsu 761-0395, Japan; kumiko.yoshihara@aist.go.jp; 3 Department of Pathology & Experimental Medicine, Graduate School of Medicine, Dentistry and Pharmaceutical Sciences, Okayama University, Okayama 700-8558, Japan; 4 National Institute of Advanced Industrial Science and Technology (AIST), Research Institute for Advanced Electronics and Photonics, Tsukuba 305-8568, Japan; kakehata-masayuki@aist.go.jp (M.K.); hidehiko.yashiro@aist.go.jp (H.Y.); 5 Advanced Research Center for Oral and Craniofacial Sciences, Okayama University Dental School, Okayama 700-8558, Japan; nagaoka@okayama-u.ac.jp

**Keywords:** zirconia, lithium disilicate, femtosecond laser, irradiation, surface roughness

## Abstract

All-ceramic restorations have become increasingly popular in dentistry. Toward ensuring that these restorations adhere to the tooth structure, this study determines the optimal femtosecond laser (FL) treatment parameters for lithium disilicate glass-ceramics and highly translucent zirconia ceramics with respect to surface morphology. For both the ceramics, the following surface conditions were investigated: (1) as-sintered; (2) Al_2_O_3_ sandblasted; (3) FL treatment (dot pattern with line distances of 14, 20, and 40 µm); (4) FL treatment (crossed-line pattern with a line distance of 20 and 40 µm). Surface roughness parameters were estimated using a 3D confocal laser microscope; microstructures were analyzed using a scanning electron microscope. Peak fluence (F_peak_) values of 4 and 8 J/cm^2^ and irradiation numbers (N) of 20 and 10 shots were selected to create dot patterns in highly translucent zirconia and lithium disilicate glass-ceramics, respectively. Furthermore, F_peak_ = 8 J/cm^2^ and N = 20 shots were chosen to obtain crossed-line patterns in both ceramics. Our results show that lithium disilicate glass-ceramics and highly translucent zirconia exhibit a similar surface morphology under each of the surface treatment conditions. Therefore, FL irradiation of dot or crossed-line patterns (at a distance of 20 and 40 µm) are potential candidates for future investigations.

## 1. Introduction

All-ceramic restorations have garnered increasing popularity in dentistry due to their superior aesthetics and biocompatibility compared to porcelain-fused-to-metal restorations. Further, lithium disilicate glass-ceramics and zirconia ceramics are the most commonly used materials for all-ceramic restorations as they satisfy the requirements pertaining to aesthetics as well as mechanical properties [1].

To achieve long-term clinical success with all-ceramic restorations, they must adhere to the tooth structure [2]. Pretreatment of the surfaces is required to ensure long-term bonding to these ceramics, particularly that of lithium disilicate glass-ceramics [3]. Conventional approaches typically employ hydrofluoric acid for the surface pretreatment of lithium disilicate glass-ceramics [4], while alumina sandblasting is used for zirconia ceramics [5]. These pretreatments, however, may reduce the strength of the ceramic materials [6,7].

The current study, thus, proposes femtosecond laser (FL) treatment as an alternative method of surface pretreatment. FL treatment is often referred to as abrasion and can be used to treat metals, silicones, and insulators without resulting in surface damage. However, the optimal FL treatment parameters for lithium disilicate glass-ceramics or highly translucent zirconia ceramics (6 mol% yttria partially stabilized zirconia: 6Y-PSZ) have not been established to date.

Thus, the purpose of this study is to ascertain the optimal FL treatment parameters for lithium disilicate glass-ceramics or highly translucent zirconia ceramics in terms of surface morphology.

## 2. Materials and Methods

### 2.1. Materials

The following two ceramics were investigated in this study: lithium disilicate glass-ceramics (IPS e.max CAD HT, Ivoclar Vivadent, Schaan, Lichtenstein) and highly translucent zirconia ceramics (6Y-PSZ: KATANA UTML, Kuraray Noritake Dental, Tokyo, Japan). Fully-sintered square-shaped specimens were prepared (14 × 14 × 2.2 mm) for each ceramic grade.

### 2.2. Pilot Experiment

To determine the optimal laser treatment settings for lithium disilicate glass-ceramics and highly translucent zirconia, an FL (Pharos, Light Conversion Inc., Vilnius, Lithuania) was employed, and the following laser parameters were investigated: (1) Three different peak fluences (F_peak_ = 2, 4, 8 J/cm^2^) and four different irradiation numbers (N = 5, 10, 20, 40 shots) were investigated for dot pattern processing; (2) four different peak fluences (F_peak_ = 2, 4, 8,10 J/cm^2^) and four equivalent irradiation numbers (N_eq_ = 5, 10, 20, 40 shots) were investigated for attaining cross-line patterns. The peak fluence is equal to the peak energy irradiated by the Gaussian laser beam, as defined as follows [8]:

F_peak_ = 2E/(πr_0_^2^), (1)

where E is the laser pulse energy and r_0_ is the radius of the irradiated beam at 1/e^2^ intensity on the surface of specimens (r_0_ = 16.1 µm). The equivalent irradiation number for line scan irradiation, defined as the number of pulses irradiated onto a single point, is given as [9]:

N_eq_ = 2r_0_ f_rep_/v_scan_, (2)

where f_rep_ and v_scan_ denote the laser pulse repetition frequency and the laser beam scanning speed, respectively.

### 2.3. Surface Treatments

Based on the results of the pilot experiment, the following surface conditions were investigated: (1) as-sintered: specimens were preserved untreated on the surface (negative control); (2) Al_2_O_3_ sandblasted: the specimens were sandblasted for 10 s at 0.2 MPa using 50-µm-thick alumina (Heraeus Kulzer, Hanau, Germany) (positive control); (3) FL treatment (dot pattern): the specimens were irradiated with three different dot patterns (line distances of 14, 20, and 40 µm) using an FL. (4) FL treatment (crossed-line pattern): the specimens were irradiated with two different crossed-line patterns (line distances of 20 and 40 µm) using an FL. The details of the FL treatment settings are summarized in Table 1 and Table 2. The three line distances for the dot patterns were selected because the 20-µm diameter dots cover the entire surface with the 14-µm line distance, the dots overlap slightly with the 20-µm line distance, and the dots clearly separate with the 40-µm line distance. Meanwhile, the line distances for the crossed-line patterns were selected because the 20-µm width lines slightly overlap for the 20-µm line distance, and the lines clearly separate for the 40-µm line distance.

### 2.4. Surface Roughness

The surface roughness was determined at a magnification of 50× using a 3D confocal laser microscope (LEXT OLS4100, Olympus, Tokyo, Japan). Additionally, the surface roughness parameters (*S_a_* and *R_a_*) were calculated using the ProfilmOnline software (Filmetrics, San Diego, CA, USA) with an 80-µm cutoff value.

### 2.5. Microstructural Analysis

Scanning electron microscopy was used to analyze the microstructures. Prior to SEM observation, the specimen surfaces were treated using an osmium coater (Neoc-STB, Meiwafosis, Tokyo, Japan). Subsequently, the specimen surfaces were examined using a field emission gun-SEM (FEG-SEM, JSM-6701F, JEOL, Tokyo, Japan) at 5 kV.

## 3. Results

### 3.1. Pilot Experiment

**Figure 1:**
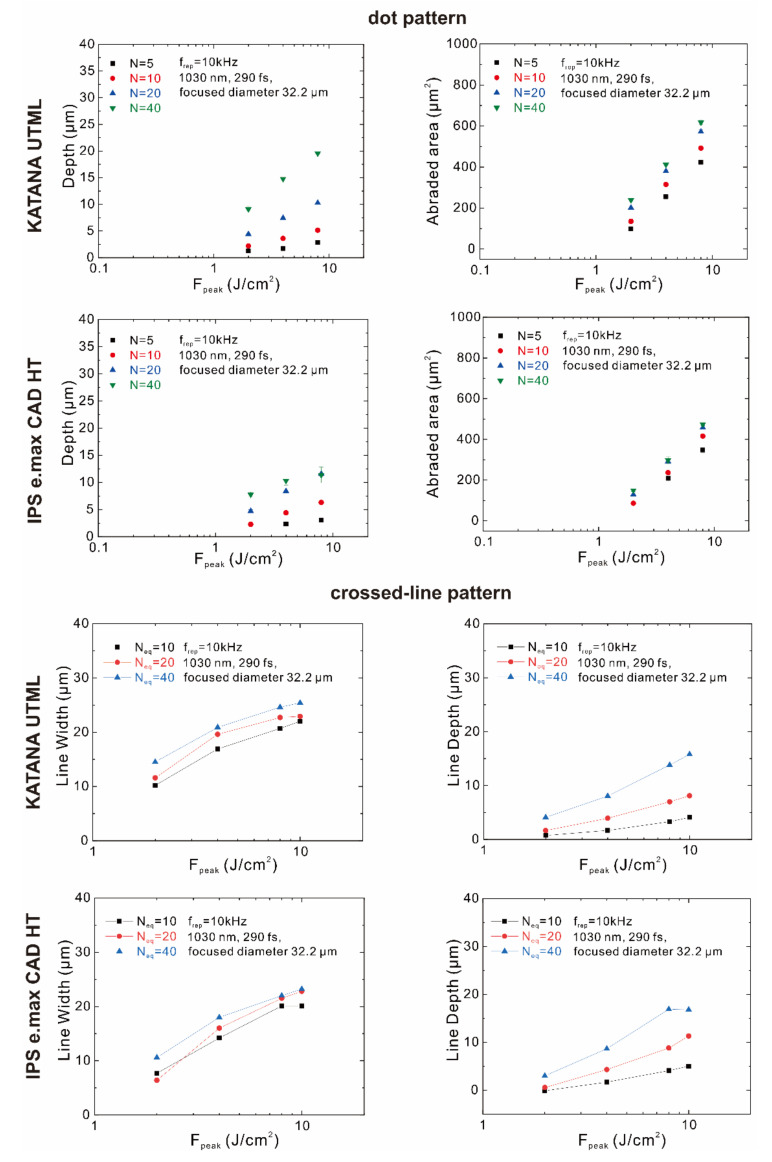
Relationship between the peak fluence and treated area or depth for the dot and line pattern processing.

The relationship between the peak fluence and the treated area or depth is summarized in Figure 1 for both the dot and line pattern processing. The ablated profiles depend on both the F_peak_ and the number of irradiated laser pulses. The irradiation parameters listed in Table 1 were selected to make the ablated area and depth similar for the two ceramics: the ablated diameter and depth were around 20 µm and 7 µm, respectively. For the dot pattern processing, a peak fluence of 8 J/cm^2^ with 10 shots resulted in a treated area of approximately 420 µm^2^ for the lithium disilicate glass-ceramics, whereas a peak fluence of 4 J/cm^2^ with 20 shots resulted in a treated area of approximately 400 µm^2^ for the highly translucent zirconia. Additionally, the treated depth was observed to be 6.3 µm harboring a peak fluence of 8 J/cm^2^ with 10 shots for the lithium disilicate glass-ceramics. On the other hand, the treated depth for the highly translucent zirconia was 7.4 µm with a peak fluence of 4 J/cm^2^ and 20 shots. The total energy input per unit area (F_peak_ × N) was set to the same value (80 J/cm^2^) for the two ceramics.

For the line pattern processing, the irradiation parameters listed in Table 2 were selected to make the ablated linewidth and depth similar for the two ceramics: the ablated width and depth were around 20 µm and 7 µm, respectively. Furthermore, a peak fluence of 8 J/cm^2^ with the equivalent irradiation of 20 shots was selected for both ceramics, which produced a linewidth of 21.5 µm and a depth of 8.8 µm for the lithium disilicate glass-ceramics, and a linewidth of 22.7 µm and a depth of 7.1 µm for the highly translucent zirconia.

Using the obtained data, the FL treatment parameters are selected and summarized in Table 1 and Table 2.

### 3.2. Surface Roughness

**Figure 2:**
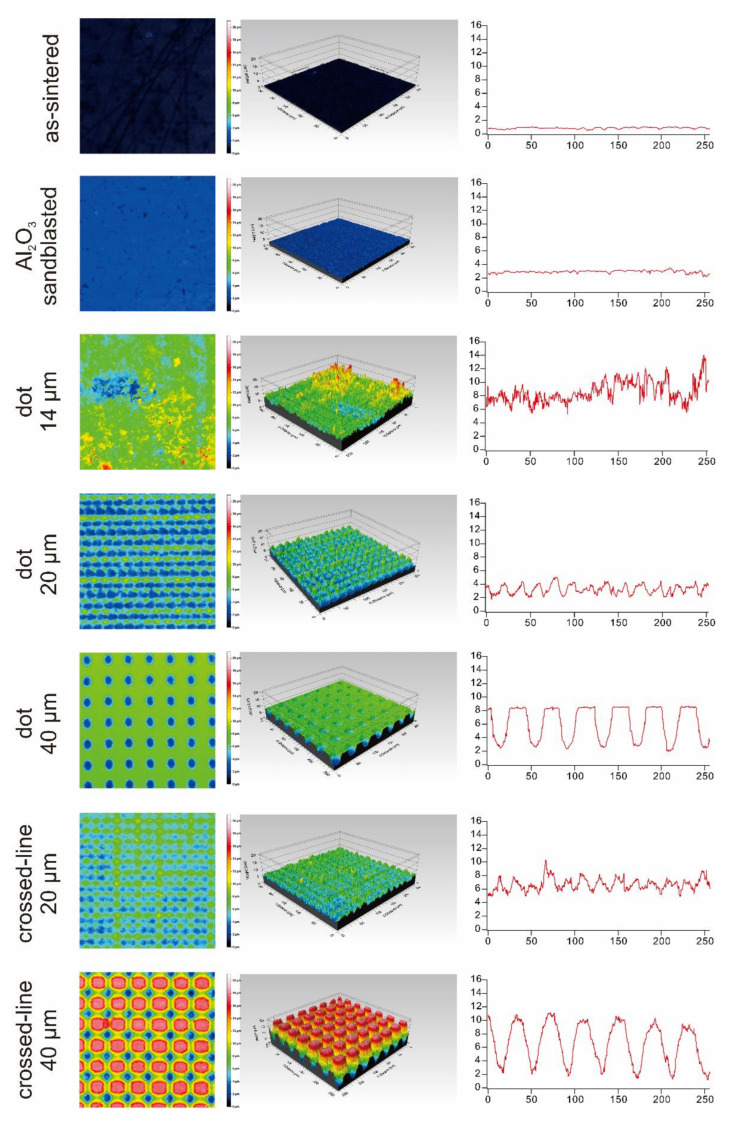
Summary of the surface roughness analysis for lithium disilicate glass-ceramics.

**Figure 3:**
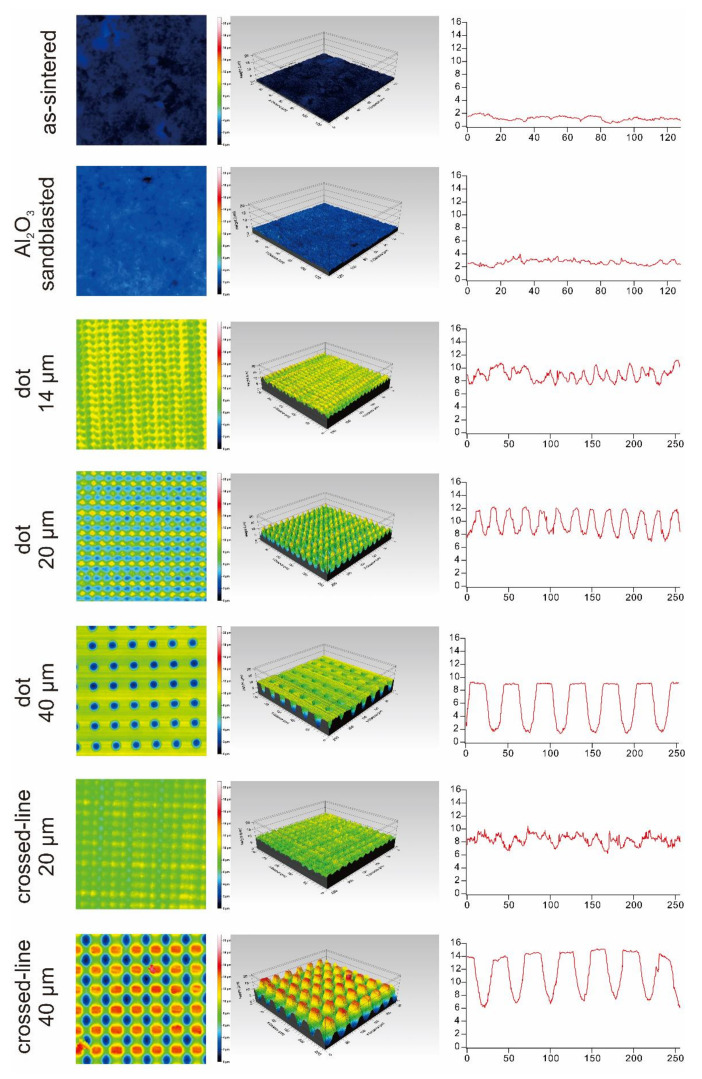
Summary of the surface roughness analysis of highly translucent zirconia ceramics.

Figure 2 and Figure 3, and Table 3 summarize the surface roughness of the surface-treated lithium disilicate glass-ceramics and highly translucent zirconia. Using the pilot test, it was determined that the surface roughness of both of these ceramics is comparable for each surface treatment condition.

### 3.3. Microstructural Analysis

**Figure 4:**
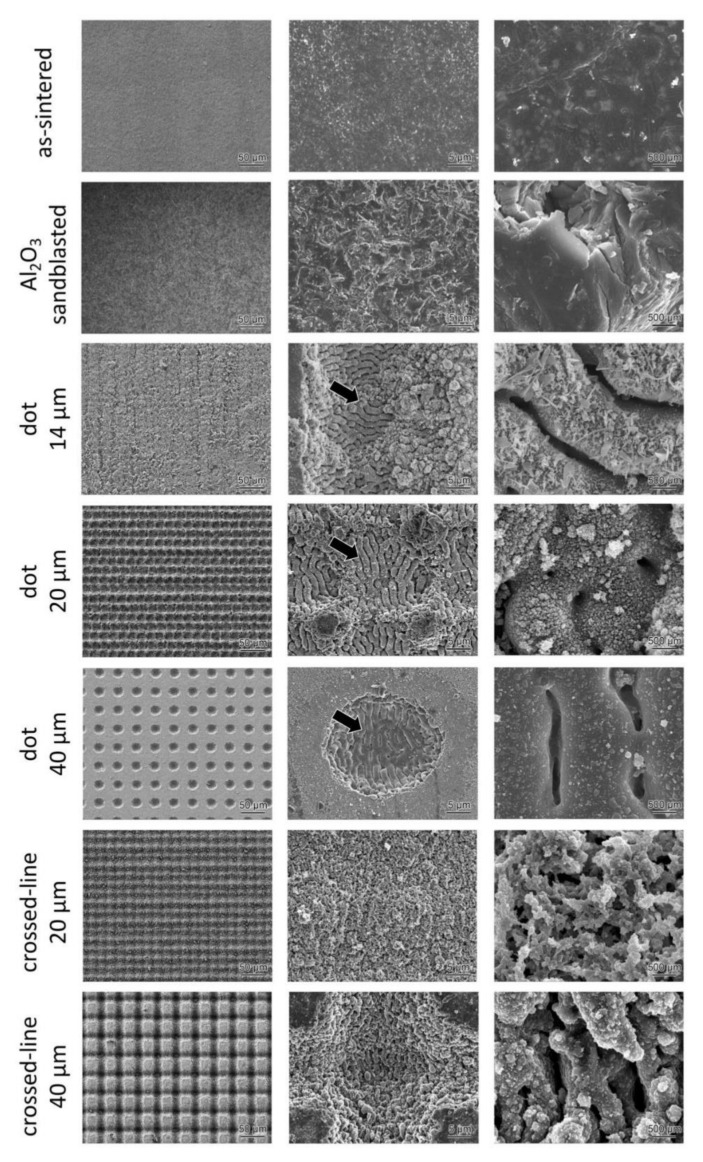
Microstructural analysis of the surface-treated lithium disilicate glass-ceramics. Clear LIPSS can be observed (black arrows) in the three types of dot patterns.

**Figure 5:**
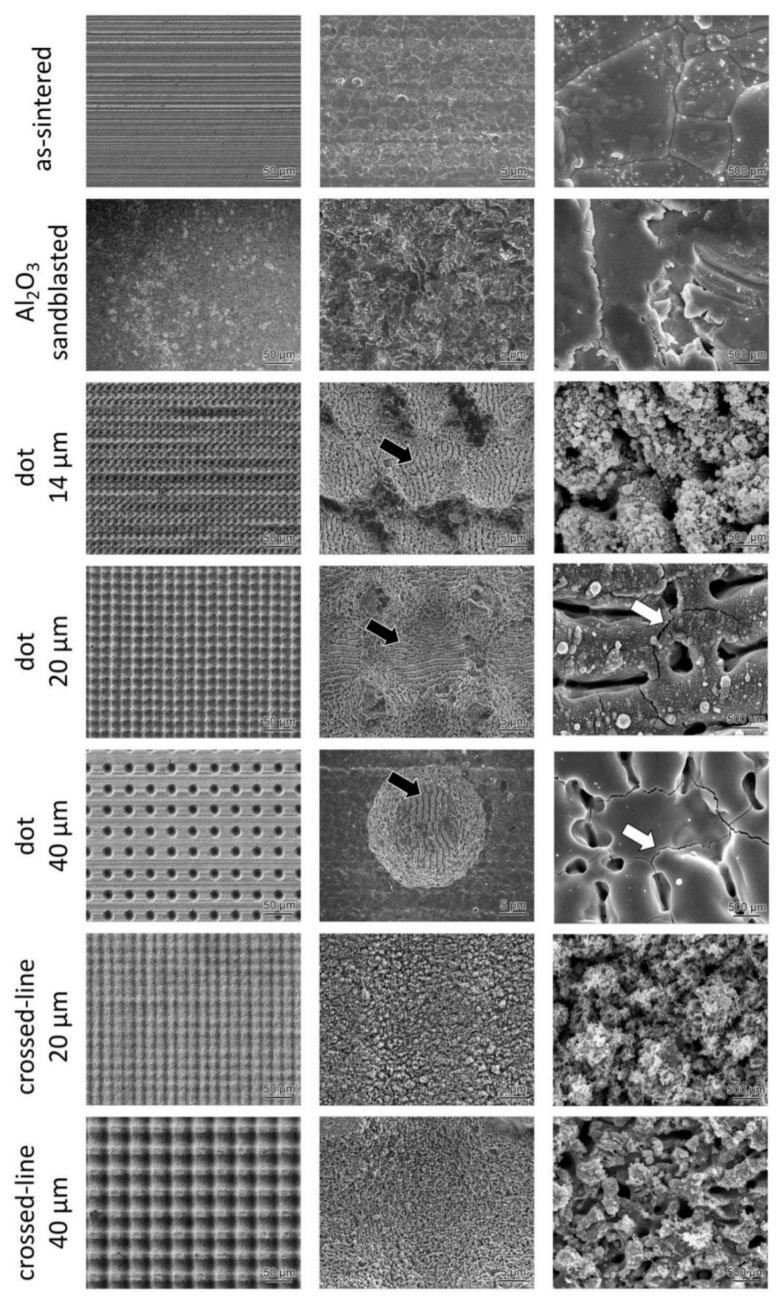
Microstructural analysis of the surface-treated highly translucent zirconia ceramics. In the three types of dot patterns, clear LIPSS (black arrows) as well as micro-cracks (white arrows), can be observed.

The microstructural analysis conducted using an SEM revealed no microcracks in the lithium disilicate glass-ceramic surfaces irradiated with an FL (Figure 4). On the other hand, microcracks could be observed in the highly translucent zirconia surfaces irradiated with an FL (Figure 5, white arrows). Additionally, for both ceramic grades, FL irradiation with a dot pattern revealed distinct laser-induced periodic surface structures (LIPSS), whereas the same was not observed in specimens irradiated with crossed-line FL patterns. Using the KATANA UTML (4 J/cm^2^, 20 shots), the LIPSS period was measured to be 1.14 µm, whereas the IPS e.max CAD HT calculated it to be (8 J/cm^2^, 10 shots) 1.6 µm.

## 4. Discussion

The effect of FL irradiation on lithium disilicate glass-ceramics and highly translucent zirconia was investigated in this study. Our findings indicate that laser-irradiated surfaces exhibit a higher degree of surface roughness than sandblasted surfaces. Additionally, the SEM images demonstrate the formation of LIPSS and microcracks on femtosecond laser-irradiated ceramic surfaces.

Prior to the actual FL irradiation, we conducted a pilot experiment to determine the laser’s actual setting. To obtain structures similar to those of lithium disilicate glass-ceramics and highly translucent zirconia, we used the following FL settings in accordance with a previous study [10]: f_rep_ = 10 kHz, wavelength = 1030 nm, pulse width = 290 fs; for KATANA UTML: F_peak_ = 4 J/cm^2^, N = 20 shots; for IPS e.max CAD HT: F_peak_ = 8 J/cm^2^, N = 10 shots, for dot-pattern processing and F_peak_ = 8 J/cm^2^, N_eq_ = 20 shots for both KATANA UTML and IPS e.max HT for crossed-line pattern processing.

We investigated the surface roughness in the present study using a 3D confocal laser microscope. Three-dimensional confocal laser microscopy is a non-contact technique applied for determining the roughness of a surface. Both lithium disilicate glass ceramics and highly translucent zirconia exhibit rougher surfaces when irradiated with laser compared to sintering or sandblasting. Our findings corroborate those of Okutan et al., who demonstrated that the surface roughness, *R_a_*, of laser-irradiated surfaces is significantly greater than that of as-sintered or sandblasted surfaces [11]. Garcia-Sanz et al. investigated the surface roughness differences between sintered zirconia, Al_2_O_3_ sandblasted zirconia, and FL-irradiated zirconia [12]. Accordingly, the surface roughness, *R_a_*, of zirconia surfaces irradiated with an FL was significantly greater than that of sintered and the Al_2_O_3_ sandblasted surfaces.

SEM images revealed the presence of clear LIPSS on the FL-irradiated ceramic surfaces with dot patterns. The reason that the vertical and horizontal orientations of LIPSS are different in some images is that the sample orientation was rotated by 90 degrees during observation. LIPSS, on the other hand, was not observed in FL-irradiated ceramics with crossed-line patterns. This type of LIPSS is typically observed in the high fluence region (F ≥ 1 J/cm^2^), which can be partially attributed to the melting of the surface followed by re-solidification. Kakehata et al., described the formation of LIPSS on 3Y-TZP for the first time [13]. The LIPSS produced by the FL on 3Y-TZP was comparable to that produced on the highly translucent zirconia ceramics (6Y-PSZ) in this study. The LIPSS lines were parallel to the laser beam’s polarization and had a period equal to or slightly greater than the laser’s wavelength. Thus, the LIPSS period is generally dependent on the fluency and shot count. An increase in fluence resulted in an increase in the LIPSS period. When a spatially Gaussian beam is used to create crossed-line patterns, some parts of the lines are irradiated by the Gaussian beam’s low-fluence trailing edge (F ≤ 1 J/cm^2^). Even if the LIPSS is formed by the scanning beam’s center, irradiation of the trailing edge of the Gaussian beam associated with the scanning results in LIPSS ablation; this is why the LIPSS observed in dot patterns is not visible in the line scanned samples. Additionally, as demonstrated by the SEM images, the LIPSS contained microcracks, which may reduce the strength of the ceramics [14].

Numerous studies have been conducted to determine the effect of femtosecond laser irradiation on dental zirconia ceramics [11,12,15,16,17,18,19,20,21]. Tzanakakis et al., investigated the shear bond strength of femtosecond laser-irradiated highly translucency zirconia (5Y-PSZ) to composite cement [19]. They concluded that the FL treatment of 5Y-PSZ ceramics is a viable alternative for mechanical cement retention. Okutan et al. examined the effect of FL treatment and/or sandblasting protocols on the surface roughness, *R_a_*, and shear bond strength of zirconia to composite cement [11]. They concluded that the FL treatment of pre-sintered zirconia resulted in a stronger bond compared to the other conditions. Kakehata et al. investigated the flexural strength of 3Y-TZP zirconia grades subsequent to FL irradiation [17]. They found that specimens treated with FL had lower flexural strength than the untreated control specimens.

Yavuz et al. reported that the FL treatment of lithium disilicate glass-ceramics improved the bond strength to adhesive resin cement [22]. They reported that the highest shear bond strength values were found in lithium disilicate glass-ceramics that had been etched with hydrofluoric acid or sandblasted with a tribochemical silica coating. While the FL treatment resulted in a lower bond strength than hydrofluoric acid etching or tribochemical silica sandblasting, the shear bond strength of the FL-treated lithium disilicate glass-ceramics was greater than that of Al_2_O_3_ sandblasted lithium disilicate glass-ceramics. Graf et al. examined the formation of LIPSS on three distinct glass-ceramic specimens [23]. They discovered that soda-lime-silicate glass exhibited a different LIPSS than fused silica and borosilicate glass. On the other hand, no study has been conducted on the effect of FL surface treatment on the mechanical strength of lithium disilicate glass-ceramics.

In the present study, we have exclusively focused on the surface morphology of sandblasted and FL-irradiated lithium disilicate glass-ceramics and the highly translucent zirconia grade. However, when considering FL irradiation as a surface pretreatment, it is necessary to consider not only the morphological factors but also the mechanical properties. Moreover, laser-induced phase transformation may influence the longevity of ceramic-based restorations made of highly translucent zirconia. Further research is necessary to determine the effect of FL irradiation on the crystallography, mechanical properties, and bond strength of lithium disilicate glass-ceramics and highly translucent zirconia grades. In addition, the bond strength between two ceramics and composite cement with FL-irradiated surfaces should be investigated.

## 5. Conclusions

In summary, the optimal FL settings were f_rep_ = 10 kHz, wavelength = 1030 nm, and pulse width = 290 fs for the highly translucent zirconia ceramics and lithium disilicate glass-ceramics. F_peak_ values of 4 J/cm^2^ and 8 J/cm^2^ and N values of 20 and 10 shots were deemed optimal for the dot-pattern processing of highly translucent zirconia ceramics and lithium disilicate glass-ceramics, respectively. Moreover, an F_peak_ of 8 J/cm^2^ and a N_eq_ of 20 shots were deemed optimal for the crossed-line pattern processing in both translucent zirconia and lithium disilicate glass-ceramics. Under these settings, the FL-irradiated surfaces of lithium disilicate glass-ceramics and highly translucent zirconia (6Y-PSZ) exhibited similar morphologies in these conditions. Accordingly, FL irradiation of dot or crossed-line patterns at a distance of 20 µm and 40 µm were selected as potential candidates for future investigations. Using these optimal settings, we will further investigate the influence of FL irradiation on the crystallography, mechanical properties, and bond strength of lithium disilicate glass-ceramics and highly translucent zirconia grades. Consequently, the potential clinical application of FL irradiation in surface treatment will be analyzed.

## Figures and Tables

**Table 1:** Details of the femtosecond laser (FL) irradiation settings for dot pattern processing.

Parameters ^1^	IPS e.max CAD HT	KATANA UTML
f_rep_, wavelength, pulse width	10 kHz, 1030 nm, 290 fs	10 kHz, 1030 nm, 290 fs
F_peak_	8 J/cm^2^	4 J/cm^2^
N	10 shots	20 shots
Ablated dot diameter(horizontal × vertical)	25.5 µm × 22.3 µm	21.3 µm × 23.6 µm
Ablated depth	6.3 µm	7.4 µm

^1^ f_rep_: pulse repetition frequency; F_peak_: peak fluence; N: the number of shots.

**Table 2:** Details of the femtosecond laser irradiation settings for a crossed-line pattern processing.

Parameters ^1^	IPS e.max CAD HT	KATANA UTML
f_rep_, wavelength, pulse width	10 kHz, 1030 nm, 290 fs	10 kHz, 1030 nm, 290 fs
F_peak_	8 J/cm^2^	8 J/cm^2^
N_eq_	20 shots	20 shots
Ablated width	21.5 µm	22.7 µm
Ablated depth	8.8 µm	7.1 µm

^1^ F_peak_: peak fluence; N_eq_: equivalent irradiation number.

**Table 3:** Summary of the surface roughness analysis.

Surface Condition	IPS e.max CAD HT		KATANA UTML	
	*S_a_* (µm)	*R_a_* (µm)	*S_a_* (µm)	*R_a_* (µm)
as-sintered	0.1016	0.07066	0.2098	0.05916
Al_2_O_3_ sandblasted	0.1801	0.07605	0.3227	0.109
dot 14 µm	1.336	0.7375	0.8179	0.6564
dot 20 µm	1.303	0.569	1.692	1.395
dot 40 µm	1.549	2.13	1.502	2.636
crossed-line 20 µm	0.9738	0.6644	0.7263	0.5665
crossed-line 40 µm	4.108	2.335	3.037	2.43

## Data Availability

The data presented in this study are available from the corresponding author, M.I., upon reasonable request.
